# THE PROBLEM OF INTEGRATING OF BIOLOGICAL AND CLINICAL MARKERS OF AGEING

**DOI:** 10.1093/geroni/igz038.325

**Published:** 2019-11-08

**Authors:** Arnold Mitnitski, Kenneth Rockwood

**Affiliations:** 1 Dalhousie University, Department of Medicine, Halifax, Nova Scotia, Canada; 2 Dalhousie University, Halifax, Nova Scotia, Canada

## Abstract

The number of potential biological markers of ageing increases dramatically especially with the development omics technologies. These biomarkers are not generally independent from each other and also related to clinical markers of aging that also could be markers of some illnesses. We discuss three ways of integrating biological and clinical markers of ageing: a frailty index (FI), indices of biological age, and a statistical distance as a measure of physiological dysregulation. We shows that FI has a strong theoretical support in the complex dynamical network model of the ageing process. The theory allows to explain why the interdependence of variables (representing the attributes of health) is essential for understanding of the basic properties both of the FI and of ageing such as a Gompertz law of mortality. Further progress in the field will go hand-in-hand with the development of new technologies that allow more data to be collected and interpreted.

